# Tolerance and Tachyphylaxis to Head Twitches Induced by the 5-HT2A Agonist 25CN-NBOH in Mice

**DOI:** 10.3389/fphar.2018.00017

**Published:** 2018-02-06

**Authors:** Tobias Buchborn, Taylor Lyons, Thomas Knöpfel

**Affiliations:** ^1^Laboratory for Neuronal Circuit Dynamics, Department of Medicine, Imperial College London, London, United Kingdom; ^2^Centre for Neurotechnology, Institute of Biomedical Engineering, Imperial College London, London, United Kingdom

**Keywords:** serotonergic hallucinogen, 25CN-NBOH, 5-HT_2A_ receptor, 5-HT_2C_ receptor, head twitch response, tachyphylaxis, tolerance, locomotion

## Abstract

The serotonin (5-HT) 2A receptor is the primary molecular target of serotonergic hallucinogens, which trigger large-scale perturbations of the cortex. Our understanding of how 5-HT_2A_ activation may cause the effects of hallucinogens has been hampered by the receptor unselectivity of most of the drugs of this class. Here we used 25CN-NBOH (N-(2-hydroxybenzyl)-2,5-dimethoxy-4-cyanophenylethylamine), a newly developed selective 5-HT_2A_ agonist, and tested it with regard to the head-twitch-response (HTR) model of 5-HT_2A_ activity and effects on locomotion. 25CN-NBOH evoked HTRs with an inverted u-shape-like dose-response curve and highest efficacy at 1.5 mg/kg, i.p. HTR occurrence peaked within 5 min after agonist injection, and exponentially decreased to half-maximal frequency at ~11 min. Thorough habituation to the experimental procedures (including handling, saline injection, and exposure to the observational boxes 1 day before the experiment) facilitated the animals' response to 25CN-NBOH. 25CN-NBOH (1.5 mg/kg, i.p.) induced HTRs were blocked by the 5-HT_2A_ antagonist ketanserin (0.75 mg/kg, 30 min pre), but not by the 5-HT_2C_ antagonist SB-242084 (0.5 mg/kg, i.p., 30 min pre). SB-242084 instead slightly increased the number of HTRs occurring at a 3.0-mg/kg dose of the agonist. Apart from HTR induction, 25CN-NBOH also modestly increased locomotor activity of the mice. Repeated once-per-day injections (1.5 mg/kg, i.p.) led to reduced occurrence of 25CN-NBOH induced HTRs. This intermediate tolerance was augmented when a second (higher) dose of the drug (3.0 mg/kg) was interspersed. Short-interval tolerance (i.e., tachyphylaxis) was observed when the drug was injected twice at intervals of 1.0 and 1.5 h at either dose tested (1.5 mg/kg and 0.75 mg/kg, respectively). Inducing ketanserin-sensitive HTRs, which are dependent on environmental valences and which show signs of tachyphylaxis and tolerance, 25CN-NBOH shares striking features common to serotonergic hallucinogens. Given its distinct *in vitro* selectivity for 5-HT_2A_ over non5-HT_2_ receptors and its behavioral dynamics, 25CN-NBOH appears to be a powerful tool for dissection of receptor-specific cortical circuit dynamics, including 5-HT_2A_ related psychoactivity.

## Introduction

The serotonin (5-HT) 2A receptor is a member of the 5-HT_2_ seven-transmembrane receptor family and is expressed in various cells and tissues across the mammalian body, with highest expression levels in the brain (see GPCR database; Regard et al., 2008). 5-HT_2A_ receptors are specifically enriched in the cerebral cortex, particularly on the apical dendrites of pyramidal cells in layer V (Weber and Andrade, 2010). By enhancing cortical glutamate release, 5-HT_2A_ receptors may modulate cognitive processes, such as working memory and attention (Williams et al., 2002; Mirjana et al., 2004). The 5-HT_2A_ receptor is considered as an important drug target, with potential implications of both agonists and antagonists in the treatment of various psychiatric conditions, including depression and anxiety (Mascher, 1967; Quesseveur et al., 2012; Buchborn et al., 2014; Carhart-Harris et al., 2016; Ross et al., 2016; Carhart-Harris and Goodwin, 2017). Blockade of 5-HT_2A_ receptors has been shown to counteract alterations of consciousness induced by serotonergic hallucinogens such as lysergic acid diethylamide (LSD) (Kraehenmann et al., 2017), and more specifically, 5-HT_2A_ receptors of layer 5 pyramidal cells are thought to be a key mediator of psychedelic activity (Vollenweider and Kometer, 2010; Muthukumaraswamy et al., 2013; Nichols, 2016). Despite the availability of relatively selective 5-HT_2A_ antagonists (see PDSP Ki database; Roth et al., 2000), our understanding of the general neurophysiology of (cortical) 5-HT_2A_ receptors is limited by the lack of highly selective 5-HT_2A_ agonists. The 4-iodo-2,5-dimethoxy-analog of amphetamine DOI, for instance, which often has been the drug of choice in animal studies related to 5-HT_2A_ functions, seems to exhibit confounded affinity for 5-HT_2_ as well as adrenergic receptors (Ray, 2010). In search of agonists with higher 5-HT_2A_ selectivity, a series of 48 compounds was recently developed based on the phenethylamine backbone shared by DOI-like serotonergic hallucinogens (Hansen et al., 2014). Amongst these, as indicated by an extensive follow-up *in vitro* receptor binding screening, the newly designed N-(2-hydroxy)benzyl substituted 4-cyano-2,5-dimethoxyphenethylamine (25CN-NBOH) turned out to be one of the most promising candidates: 25CN-NBOH is characterized by a high affinity for 5-HT_2A_ receptors (*in vitro* radioligand competition binding, av. Ki: ~1.72 nM), by a ≥ 100-fold selectivity for binding to this receptor as compared to a plethora of non-5-HT_2_ targets (including other G-protein coupled receptors, ion channels, transporters, and enzymes), and robust 5-HT_2A_ selectivity over 5-HT_2B/2C_ receptors (K_i_ *in vitro* [averaged across studies]: ~56 nM for 5-HT_2B_ and ~83 nM for 5-HT_2C_, respectively) (Hansen, 2010; Halberstadt et al., 2016; Jensen et al., 2017). Therefore, 25CN-NBOH appears advantageous over other currently used 5-HT_2A_ agonists subjected to a similarly scrutinous receptor profiling (Ray, 2010; see also Ki database).

Here, we aimed to determine the suitability of 25CN-NBOH for prospective brain imaging studies in chronic *in vivo* mouse models. To this end, we employed the head twitch response (HTR) as well as locomotion as a behavioral readout of central 5-HT_2A_ activity. We determined the dose range of 25CN-NBOH required for parenteral administration to induce central 5-HT_2A_ activation, the time-course of the central action, sensitivity to 5-HT_2A_ antagonism and experimental familiarity, a possible involvement of 5-HT_2C_ receptors in 25CN-NBOH induced HTRs, as well as potential development of tachyphylaxis and tolerance.

## Materials and methods

### Animals

All experimental procedures performed at Imperial College London were in accordance with the UK Animal Scientific Procedures Act (1986) under Home Office Personal and Project licenses (I5B5A6029, IA615553C; PPL 70/7818), following appropriate ethical review.

Adult mice of both sexes and with mixed genetic background (stock of mainly C57BL/6JxB6CBAF1 background) were bred in-house at the Central Biomedical Services (CBS) of Imperial College London. They were housed in individually ventilated cages with a 12:12 h light/dark cycle and maintained at an ambient temperature of 21 ± 2°C at 55 ± 10% humidity. Mice were provided with standard rodent-chow pellets (Special Diet Services, #RM1) and water *ad libitum*.

### Drugs

25CN-NBOH (a kind gift from Jesper L. Kristensen, University of Copenhagen) and ketanserin (Tocris Biosciences; Avonmouth, UK) were dissolved in isotonic saline, SB-242084 (Tocris Biosciences; Avonmouth, UK) in DMSO (10% in saline). Drugs were applied intraperitoneally or subcutaneously (<10 ml/kg), as indicated.

### Behavioral experiments

Animals were allowed to habituate to the (observational) non-home boxes (25.5 × 12.5 × 12.5 cm [L × W × H]) for 30 min. Observational boxes were shielded with red acrylic plates to minimize the animals' visual awareness of the experimenter.

#### Head twitches

For quantification of HTRs, animals were treated with saline or the respective dose of 25CN-NBOH (0.5–3.0 mg/kg, i.p. (or 1.5 mg/kg, s.c.]) and observed for 20–60 min. The frequency of head twitches, defined as rapid and brisk rotational movement of the head around the longitudinal axis of the animals' body (Handley and Singh, 1986) (see supplemental Movie), was counted in 2- or 5-min bins starting directly after injection. The sensitivity of 25CN-NBOH (1.5 mg/kg [vs. 3.0] mg/kg, i.p.) induced head twitches to 5-HT_2A_ and/or 5-HT_2C_ antagonism was tested by pre-treating the animals with ketanserin (0.75 mg/kg, i.p.) and SB-242084 (0.5 mg/kg, i.p.), respectively, 30 min prior to agonist administration. The impact of experimental familiarity was investigated by simulation of aspects of the experimental procedure (i.e., handling, injection of saline, and exposure to the observational non-home cages for 30 min before, and for 20 min after saline injection) 1 day before the actual 25CN-NBOH experiment.

Tachyphylaxis, defined as in-between-session tolerance occurring at repeated short-interval application, was tested by injecting saline or 25CN-NBOH (0.75 or 1.5 mg/kg, i.p.) twice at a 1.0- or 1.5-h interval, and comparing the average HTR frequency in each 30-min observation period. For subchronic tolerance, animals were treated for 4 days with saline or 25CN-NBOH. 25CN-NBOH was injected once (1.5 mg/kg, i.p. [morning]) or twice (1.5 mg/kg, i.p. [morning] and 3.0 mg/kg, i.p. [evening of days 1–3]) per day and HTRs were counted for 20 min after each morning application. The twice-per-day regimen, with the increased dose (i.e., 3.0 mg/kg) in the evening, was chosen based on findings for LSD, which indicate that tolerance development is slightly facilitated by a higher frequency of application, but more strongly facilitated by a higher frequency of application *with* increased dosing every other application (Buchborn et al., 2016).

#### Locomotion

Locomotion was quantified for 90 min directly after saline or 25CN-NBOH (1.5 mg/kg, i.p.) injection, averaged across three 30-min bins, and analyzed for distance traveled (in m), mobility time (in s), and average speed (in m/s) using Any-Maze Video Tracking Software (Stoelting). *Mobility* refers to times animals showed locomotion or rearing (excluding freezing and stationary grooming); *speed* was calculated as distance traveled against the cumulative mobility time periods.

### Statistics

The data from the dose-response, antagonist, and pre-habituation experiments were analyzed using non-parametric Kruskal-Wallis test with Dunn's multiple *post-hoc* comparisons, or Mann-Whitney U-testing (as a priori planned). Tolerance (HTR) and time-course data (HTR and locomotion) were subjected to a two- and three-factor ANOVA with repeated measures on one factor, respectively, and followed up on by Bonferroni-corrected multiple comparisons. For HTRs, time-course data of interest were fitted by non-linear regression analysis, and the decay constant provided by the best-fit exponential equation (HTR_t =_ ${\text{HTR}}_{0}^{*}$ e^−lambda*t^) subsequently used for determination of 25CN-NBOH's half-life (t½ = ln[2]/lambda). Calculations were carried out by SPSS or GraphPad Prism software. Statistical significance was assumed if the null hypothesis (e.g., drug has no action) could be rejected at ≤ 0. 05 probability level.

## Results

### Dose-response relationship, ketanserin-sensitivity, and effect of experimental familiarity on 25CN-NBOH induced head twitches

**Figure 1:**
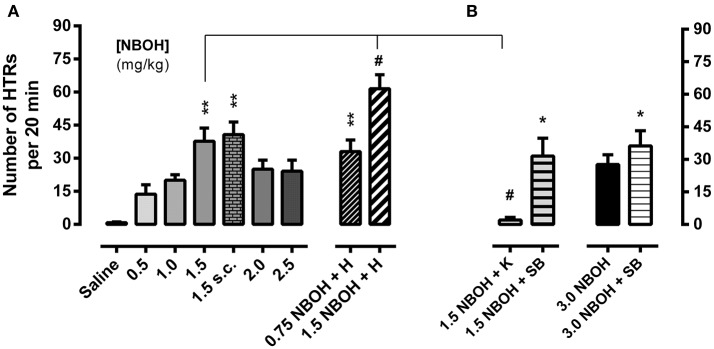
25CN-NBOH induced head twitches (HTRs) (as observed for the first 20 min post-application). **(A)** Dose–response relation (0.5–2.5 mg/kg i.p. vs. 1.5 mg/kg s.c.) and effect of experimental habituation (0.75 vs. 1.5 mg/kg NBOH i.p. + H). **(B)** Effect of the 5-HT_2A_ antagonist ketanserin [1.5 mg/kg NBOH + 0.75 mg/kg K (30 min pre-treatment), i.p.] and the 5-HT_2C_ antagonist SB-242084 [1.5 vs. 3.0 mg/kg NBOH + 0.5 mg/kg SB (30 min pre-treatment), i.p.], respectively. Mean + SEM; *n* = 4–6 per group. Kruskal-Wallis, *post-hoc* comparison to saline, ^*^p ≤ 05 and ^**^p ≤ 01; or to agonist w/o habituation and w/o antagonist pre-treatment, respectively, #*p* ≤.05.

Mice received a single injection of either saline (control) or 25CN-NBOH (0.5–3.0 mg/kg, [i.p. or s.c.]) and were monitored for HTRs during the following 20 min. While control animals in this observation period rarely showed any behaviors rated as HTRs [0.83 ± 0.31 (mean ± SEM)], the incidence of HTRs markedly increased for all tested doses of 25CN-NBOH. Frequencies (mean ± SEM) ranged from 13.67 ± 4.26 for the lowest dose (0.5 mg/kg, i.p.), 37.67 ± 6.04 to 40.67 ± 5.77 for the medium dose (1.5 mg/kg, i.p. vs. s.c.), and 27.60 ± 4.52 for the highest dose tested (3.0 mg/kg, i.p.; Figures 1A,**B**). Inference-statistically, however, only the 1.5-mg/kg dose significantly differed from the saline group (X^2^ [10, *N* = 62] = 31.49, *p* ≤ 0.001; Dunn's *post-hoc* [comparison to control], 1.5 mg/kg i.p. or s.c., each *p* ≤ 0.01). At none of the doses tested, stereotypies other than HTRs (including any symptoms of the serotonin syndrome) were noted.

The 5-HT_2A_ antagonist ketanserin (0.75 mg/kg, i.p.), when applied 30 min before the agonist, near-completely prevented the 25CN-NBOH (1.5-mg/kg, i.p.) induced HTRs (mean ± SEM: 2.0 ± 1.29) (X^2^ [4, *N* = 30] = 14.47, *p* ≤ 0.01; Dunn's *post-hoc* [comparison to 1.5 NBOH w/o antagonist and to control, respectively], *p* ≤ 0.05 and n.s.). The selective 5-HT_2C_ antagonist SB-242084 (0.5 mg/kg, i.p., 30 min pre-treatment), did not prevent 25CN-NBOH from inducing significant HTRs (mean ± SEM: 31.50 ± 8.25; Dunn's *post-hoc* (comparison to saline), *p* ≤ 0.05]. SB-242084, although not affecting the effect of 1.5-mg/kg 25CN-NBOH, increased the animals' response to the 3.0-mg/kg dose of the agonist, leading to HTRs of significant extent (mean ± SEM: 36.20 ± 7.02, *p* ≤ 0.05; Figure 1B).

Handling the animals and thoroughly habituating them to the experimental procedures (including exposure to observational boxes and saline injection 1 day before the 25CN-NBOH experiment), respectively, facilitated agonist induced HTRs; a 0.75-mg/kg *sub-threshold* dose was enabled to induce significant twitching (mean ± SEM: 33.0 ± 5.28; *p* ≤ 0.01) and the response to a 1.5-mg/kg dose was significantly increased (mean ± SEM: 61.5 ± 6.3; *p* ≤ 0.05; Figure 1A).

### Time-course of 25CN-NBOH induced head twitches

**Figure 2:**
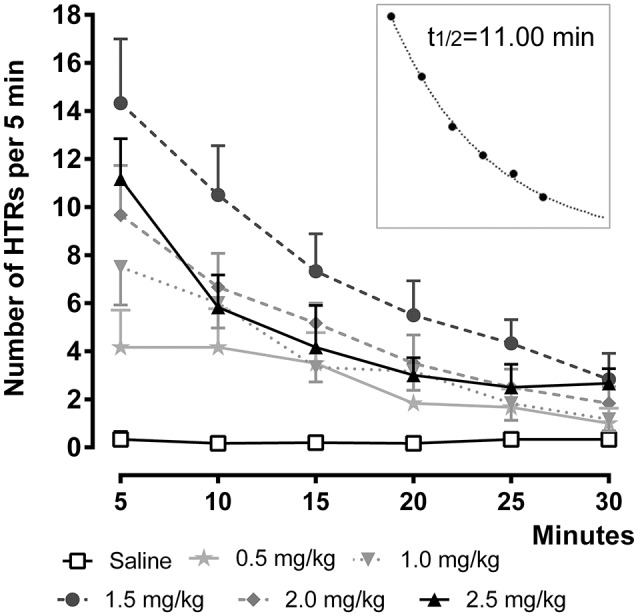
Time-course of 25CN-NBOH (0.5–2.5 mg/kg, i.p.) induced head twitches (HTRs) (as observed for the first 30 min post-application). Mean ± SEM; *n* = 6 per group. The inset replots the means of the 1.5-mg/kg dose as fitted by an exponential decay function.

To investigate the time-course of the behavioral effect of the different 25CN-NBOH doses, we counted HTRs in 5-min bins as they occurred over 30 min post-injection (Figure 2). Repeated measures ANOVA with Bonferroni-corrected *post-hoc* analysis revealed significant increases in HTR frequency as a function of time [*F*_(1.84, 27.66)_ = 57.52, *p* ≤ 0.001], treatment [*F*_(2, 15)_ = 30.41, *p* ≤ 0.001], as well as of time × treatment interaction [*F*_(3.68, 27.66)_ = 26.52, *p* ≤ 0.001]. All but the 0.5- and the 1.0-mg/kg dose elicited significant HTRs in the first observation window (0–5 min), where they peaked (1.5 mg/kg: 14.33 ± 2.67, *p* ≤ 0.001; 2.0 mg/kg: 9.67 ± 2.6, *p* ≤ 0.05; 2.5 mg/kg: 11.17 ± 1.68, *p* ≤ 0.01) and then steadily decreased over time. As depicted in the inset of Figure 2, the temporal course of 25CN-NBOH (1.5 mg/kg, i.p.) induced HTRs is well-fitted by an exponential decay function, with the effect of the drug declining to half after 11 min. The 1.0-, 2.0-, and 2.5-mg/kg doses decayed with similar half-lives between around 9 and 12 min.

In an additional set of experiments, we studied the time-course of the effect produced by the 1.5-mg/kg dose over a longer observation time period. To this end, 25CN-NBOH induced head twitches (i.p. vs. s.c.) were counted over 60 min at a 2-min-observation resolution. After i.p. injection, the frequency of HTRs peaked within the first 2-min bin (mean ± SEM: 6.8 ± 1.32) and then decayed with a t½ of 8.56 min. For s.c. injection, the peak occurred with a slight delay in the second 2-min bin (mean ± SEM: 7.00 ± 0.71) and decayed at a t½ of 13.59 min. Overall, as indicated by repeated measures ANOVA, there were no route-dependent time-course differences. The average (± SEM) of the three half-lives calculated for the 1.5-mg/kg dose is 11.05 ± 1.45.

### 25CN-NBOH's effect on locomotor activity

**Figure 3:**
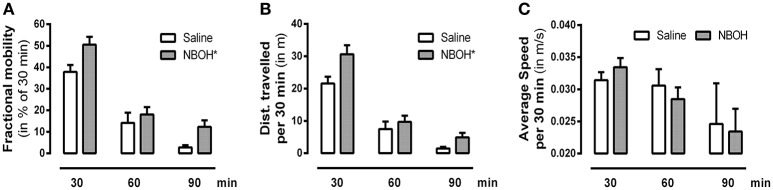
Effect of 25CN-NBOH (1.5 mg/kg, i.p.) on locomotion. Three 30-min intervals for the first 90 min after injection. **(A)** Fractional mobility (in % of 30 min); **(B)** distance traveled (in m per 30 min); **(C)** average speed (in m/s per 30 min). Mean + SEM; *n* = 8 per group. Repeated measures ANOVA, Bonferroni-corrected across-time *post-hoc* comparison to control, ^*^p ≤ 05.

To investigate how the dose of 25CN-NBOH that is the most effective in the head-twitch model (i.e., 1.5 mg/kg) would affect the animals' overall behavior, we also monitored locomotion. Splitting the 90-min observational period into three bins of 30 min, we found significant main effects for locomotion [*F*_(1, 14)_ = 571.22, *p* ≤ 0.001], treatment [*F*_(1, 14)_ = 4.62, *p* ≤ 0.05], time [*F*_(2, 28)_ = 140.97, *p* ≤ 0.001], as well as significant interactions in terms of locomotion × treatment [*F*_(1, 14)_ = 5.44, *p* ≤ 0.05] and locomotion × time [*F*_(1.71, 23.98)_ = 111.17, *p* ≤ 0.001]. Averaged across the three time-bins, 25CN-NBOH treated animals were more mobile [mean ± SEM (% of 30 min): 18.23 ± 2.75 (saline) vs. 26.93 ± 2.50 (NBOH), *post-hoc p* ≤ 0.05] and traveled a significantly greater distance [mean ± SEM (m per 30 min): 10.17 ± 1.45 (saline) vs. 15.07 ± 1.75 (NBOH), *post-hoc p* ≤ 0.05] than saline treated mice. However, both treated and untreated mice were more often immobile than mobile (see Figures 3A,B). Also, for the times of mobility, there was no significant difference in average speed [mean ± SEM (m/s per 30 min): 0.029 ± 0.002 (saline) 0.028 ± 0.002 (NBOH), *post-hoc*, n.s.] (Figure 3C). Following up on the locomotion × time interaction, both groups showed a constant decrease in the locomotion parameter *distance traveled* (mean ± SEM [m]: 21.56 ± 2.11, 7.48 ± 2.31, and 1.48 ± 0.51 [saline]; 30.62 ± 2.82, 9.67 ± 1.94, and 4.91 ± 1.37 [NBOH]) and *mobility time* (mean ± SEM [% of 30 min]: 37.86 ± 3.18, 14.12 ± 4.75, and 2.74 ± 1.03 [saline]; 50.46 ± 3.69, 18.04 ± 3.43, and 12.29 ± 2.98 [NBOH]) from the first, to the second, to the third interval (Figures 3A,**B**). For either parameter, the differences between the three factor levels of *tim*e across groups became significant (*post-hoc*, each *p* ≤ 0.05). However, no significant time-dependency could be seen for average speed (mean ± SEM [m/s per 30 min]: 0.031 ± 0.001, 0.030 ± 0.002, and 0.024 ± 0.006 [saline]; 0.033 ± 0.001, 0.028 ± 0.002, and 0.023 ± 0.003 [NBOH]) (*post-hoc* across groups, n.s.) (Figure 3C).

### Tachyphylaxis and tolerance to 25CN-NBOH induced head twitches

**Figure 4:**
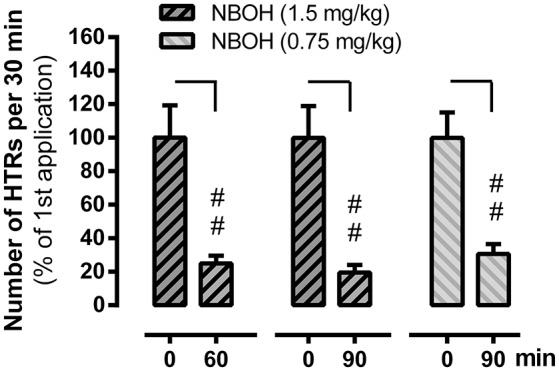
Tachyphylaxis of 25CN-NBOH (0.75 or 1.5 mg/kg, s.c.) induced head twitches (HTRs) (as observed for the first 30 min post-application). Agonist was applied twice at an interval of 60 and 90 min, respectively. Mean + SEM; *n* = 5–6 per group. Repeated measures ANOVA, Bonferroni-corrected *post-hoc* comparison to respective first application (0 min), ##*p* ≤ 01.

In an independent set of experiments, we explored whether 25CN-NBOH induced HTRs showed signs of short-term tolerance. We therefore repeatedly applied the drug at different intervals. Similarly, as reported above, 25CN-NBOH (1.5 mg/kg, s.c.) treated mice exhibited an average of 34.67 ± 4.65 HTRs within 30 min, which was significantly more than observed in saline treated mice (mean ± SEM: 1.4 ± 0.25) (*p* ≤.01). Re-applying the same dose of 25CN-NBOH one or 1½ h after the first application substantially reduced the efficacy of the drug. Main effects for time [*F*_(1, 24)_ = 64.28, *p* ≤ 0.001], group [*F*_(1, 19)_ = 62.1, *p* ≤ 0.001], and time × group interaction [*F*_(4, 24)_ = 10.47, *p* ≤ 0.001] proved significant. Whereas saline treated animals at both intervals, did not show significant differences from the first saline application (not shown), 25CN-NBOH treated mice—with only 24.98 ± 4.54% (1 h) and 19.48 ± 4.59% (1.5 h) of the original responsiveness—twitched significantly less (first vs. resp. second injection, *post-hoc* each *p* ≤ 0.01; Figure 4). As shown for the 1.5-h interval, a reduced but still highly significant loss of drug efficacy could likewise be demonstrated when 25CN-NBOH was injected twice at a lower dose (0.75 mg/kg, s.c.) to handling-habituated mice (mean ± SEM [% of the original responsiveness]: 30.66 ± 5.86; first vs. second injection, *p* ≤ 0.01) (Figure 4).

**Figure 5:**
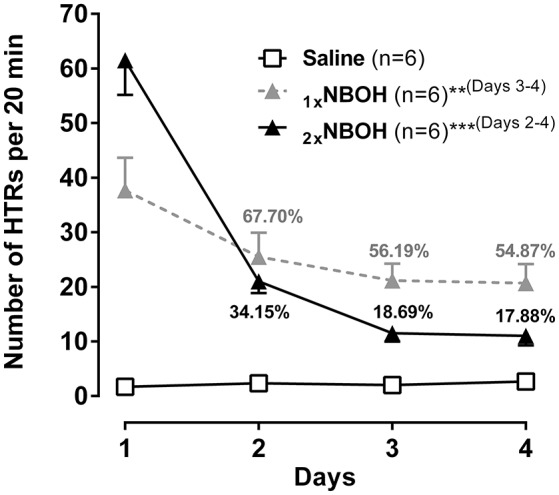
Subchronic tolerance to 25CN-NBOH induced head twitches (HTRs), with a total of four [1xNBOH: Once per day, 1.5 mg/kg (morning)] and seven [2xNBOH: Twice per day, 1.5 mg/kg (morning) vs. 3.0 mg/kg (evening)] i.p. injections, respectively. Head twitches were counted for the first 20 min after the morning application). Percent values indicate percent of HTRs relative to respective first day. Mean ± SEM; *n* = 6 per group. Repeated measures ANOVA, Bonferroni-corrected *post-hoc* comparison to respective first-day measurement, ^**^p ≤ 01, ^***^p ≤ 001 (brackets behind asterisks indicate interval(s) significance applied to).

In addition to tachyphylaxis occurring at repeated short-interval injection, a substantial loss of responsiveness to 25CN-NBOH could also be demonstrated when the drug (1.5 mg/kg, i.p.) was repeatedly applied at 1-day intervals [main effect time: *F*_(1.84, 27.66)_ = 57.51, *p* ≤ 0.001]; main effect group [*F*_(2, 15)_ = 30.41, *p* ≤ 0.001]; time × group interaction [*F*_(3.68, 27.66)_ = 26.52, *p* ≤ 0.001] (Figure 5). Unlike with the short-interval application, however, the decrease in HTRs only with the third injection of 25CN-NBOH (i.e. on the 3rd and 4th day) became significant [1x NBOH per day: Day 1 (mean ± SEM: 37.67 ± 5.99) vs. day 3 and day 4 [56.19 and 54.87% of day 1, respectively], each *p* ≤ 0.01]. When a second (higher) dose of the agonist (3.0 mg/kg, i.p.) was applied in the evening of the first 3 days, the onset of tolerance proved significant by day 2 already [2x NBOH per day: Day 1 (mean ± SEM: 61.5 ± 6.34) vs. day 2, day 3, and day 4 (34.15, 18.69, and 17.88% of day 1, respectively), each *p* ≤ 0.001]. Also, the extent of tolerance was more pronounced. Whereas the HTRs exhibited by the once-per-day-application mice on day 4 still were statistically different from the saline-treated control animals (mean ± SEM: 20.67 ± 3.49, *p* ≤ 0.001), those in the twice-per-day mice more closely approached the control level (mean ± SEM: 11.00 ± 1.73, n.s.) (Figure 5).

## Discussion

We characterized the selective 5-HT_2A_ agonist 25CN-NBOH in terms of HTRs, a behavioral output suggested to engage (Willins and Meltzer, 1997; González-Maeso et al., 2007), predict and/or model 5-HT_2A_ receptor function in the cortex (Goodwin et al., 1984; Zhang and Marek, 2008; e.g., Buchborn et al., 2015).

25CN-NBOH evoked marked HTRs in mice at the full range of doses tested (i.e., 0.5–3.0 mg/kg). In the context of the multiple-comparisons analysis, however, only the medium dose (1.5 mg/kg) turned out significant, which mirrors previous findings of the drug being most active at around 1.0–3.0 mg/kg (Fantegrossi et al., 2015; Halberstadt et al., 2016). The inverted u-shape-like dose-response curve of 25CN-NBOH (with higher doses entailing a relative loss of responsiveness) might be due to increasing occupancy of 5-HT_2C_ receptors (whose activity may counteract the 5-HT_2A_–mediated HTR induction), and/or due to 5-HT_2_ receptors requiring a tuned level of occupancy (compare for DOM, DOB, and DOI: Fantegrossi et al., 2010; Serafine and France, 2014; Buchborn et al., 2015). Consistent with its sensitivity to MDL100907 (Fantegrossi et al., 2015), one of the most selective 5-HT_2A_ antagonists at present (av. Ki [Ki database]: ~1.92 nM for _r_5-HT_2A_; ~112 nM for _r_5-HT_2C_), 25CN-NBOH (1.5 mg/kg, i.p.) induced HTRs were blocked by the 5-HT_2A_ antagonist ketanserin (av. Ki [Ki database]: ~1.6 nM for _r_5-HT_2A_; ~52 nM for _r_5-HT_2C_). The selective 5-HT_2C_ antagonist SB-242084 (av. Ki [Ki database]: ~2.85 for _h_5-HT_2C_; ~505 nM for _h_5-HT_2A_), on the other hand, did not affect HTRs evoked by a 1.5-mg/kg dose of 25CN-NBOH. Interstingly, it slightly increased the behavioral effect of the higher 3.0-mg/kg dose of the agonist. Thus, it might be suggested that low to medium doses of 25CN-NBOH primarily interact with 5-HT_2A_ receptors; but higher doses of 25CN-NBOH may also recruit 5-HT_2C_ receptors, thereby interfering with the actions mediated by 5-HT_2A_ receptors (compare for DOI: Fantegrossi et al., 2010). The finding that 3.0 mg/kg 25CN-NBOH given to mice reach brain concentrations that fall into the range of the agonist's *in vitro* affinity for 5-HT_2C_ receptors (Jensen et al., 2017), would be in line with such an interpretation.

25CN-NBOH induced HTRs were facilitated when animals had been thoroughly habituated to the experimental procedures on the day before the drug application. The latter finding supports observations made in rats, where the presence of familiar littermates allowed hallucinogens to evoke substantially more HTRs than when observed in isolation (Buchborn et al., 2015). The dependence of 5-HT_2A_ related behavior in rodents on what may be described as *state-environment* interaction, mirrors characteristics of human psychedelia, which is critically determined by set and setting, i.e., psychological constitution and environmental circumstances (rev. Hartogsohn, 2016).

25CN-NBOH induced HTRs occurred within <1 min after i.p. injection, reached maximal frequencies within 2–5 min, and declined to half-maximal at ~11 min (compare for 25I-NBOMe and 25I-NBMD: Halberstadt and Geyer, 2014). In contrast to the i.p. route, s.c. administration—as shown for the 1.5-mg/kg dose—was associated with a slight delay in peak and somewhat longer duration to half-maximum. 25CN-NBOH, both in plasma and brain, reaches maximum concentrations within 15 min, where it remains relatively stable until at least 30 min post-application (Jensen et al., 2017). The fast decline in the behavioral response observed here therefore does not seem to be a consequence of fast drug clearance. One possible explanation for the apparent disconnect between 25CN-NBOH's brain concentration and behavioral output is the development of acute tolerance. Indeed, DOI which shares the phenethylamine backbone of 25CN-NBOH, internalizes and desensitizes 5-HT_2A_ receptors within 15–20 min *in vitro* (Porter et al., 2001; Raote et al., 2013).

To follow up on the possibility of tolerance development, we applied 25CN-NBOH (1.5 mg/kg) twice at a 1.0- or a 1.5-h interval and found a rapid loss of responsiveness to the second injection (compare for LSD and DOI: Darmani and Gerdes, 1995; Buchborn et al., 2016). To test the hypothesis that the observed tachyphylaxis was due to substance accumulation (i.e., two single 1.5 mg/kg doses accumulating to a less active 3.0-mg/kg dose), we repeated the experiment injecting a 0.75-mg/kg dose (which after thorough animal habituation induced significant HTRs; see Figure 1A) twice at a 1.5-h interval. If the two 0.75 mg/kg doses had accumulated to form a 1.5-mg/kg dose, the second dose should have induced unchanged or more HTRs than the first. However, as the 0.75-mg/kg regimen led to a similar-degree tachyphylaxis as did the 1.5-mg/kg dose, it is unlikely that drug accumulation is the underlying cause for the rapid tolerance development. Serotonergic hallucinogens have been shown to affect locomotor activity in rodents (e.g., Cohen and Wakeley, 1968; Kabes et al., 1972; Wing et al., 1990), with 5-HT_2A_ activation in mice promoting hyperlocomotion (Halberstadt et al., 2009). 25CN-NBOH administration (1.5 mg/kg), in line with the latter, during the 1.5-h observation increased the distance traveled as well as the overall mobility duration. However, average speed of movement was unchanged. Also, 25CN-NBOH-treated mice (like the control animals) overall spent significantly less time mobile than immobile. Therefore, it appears unlikely that physical exhaustion caused the observed tachyphylaxis to 25CN-NBOH. Instead processes of 5-HT_2A_ regulation might play a role; indeed, DOI applied twice at a 4-h interval *in vivo*, desensitizes 5-HT_2A_ mediated glutamate release in the cortex (Scruggs et al., 2003). Finally, we investigated subchronic tolerance as it would develop with a once-a-day application of 25CN-NBOH (1.5 mg/kg, i.p.) over 4 days. HTRs steadily decreased (e.g., compare for DOI and 2C-T-7: Smith et al., 2014), with the most pronounced loss of responsiveness exhibited in between the first days. The extent of tolerance could be substantially augmented by applying a second (higher) daily dose of 25CN-NBOH (3.0 mg/kg, i.p.) at an ~8-h interval.

In summary, our data demonstrate that the selective 5-HT_2A_ agonist 25CN-NBOH induces a dose- and time-dependent, ketanserin-sensitive increase in HTRs in mice, which is subject to short-interval and subchronic tolerance. Future research, correlating HTRs with pharmacokinetic-/dynamic adaptations at various time points during tolerance development might provide insight as to a possible mechanism. Furthermore, making use of new technologies to image cortical activities in awake behaving mice using genetically encoded, cell-class specifically targeted voltage and calcium indicators (Carandini et al., 2015; Knöpfel et al., 2015; Song et al., 2017) might take our mechanistic understanding of the underlying processes from basic receptor pharmacology to systemic circuit dynamics.

## Author contributions

TB, TL, and TK: have conceptualized the study design; TB and TL: have collected and plotted the data; TB, TL, and TK: have interpreted the data and written the manuscript.

### Conflict of interest statement

The authors declare that the research was conducted in the absence of any commercial or financial relationships that could be construed as a potential conflict of interest.

## Supplementary material

Supplementary Movie 1 Head twitch response (HTR) induced by a 1.5-mg/kg i.p. dose of 25CN-NBOH (recorded at 100 Hz). The arrow indicates on- and offset of the head twitch at ~13 s. The twitch is recapitulated by a slow-motion picture-in-picture replay on the right side of the video. Format: Mp4, H.264+AAC, 720p. Duration: ~20 s.
